# Promoting healthy cooking patterns in China: Analysis of consumer clusters and the evolution of cooking pattern trends

**DOI:** 10.1371/journal.pone.0293919

**Published:** 2023-11-15

**Authors:** Chuan Bo Liang, Bin Cui, Fu Rong Wang, Jing Peng, Jian Ying Ma, Mei Yin Xu, Jun Ke, Yi Tian, Zi Qi Cui

**Affiliations:** 1 Yangzhou Polytechnic Institute, Yangzhou, Jiangsu Province, China; 2 Business School of Yangzhou University, Yangzhou, Jiangsu Province, China; 3 School of Tourism and Cuisine of Yangzhou University, Yangzhou, Jiangsu Province, China; 4 School of Public Health of Yangzhou University, Yangzhou, Jiangsu Province, China; 5 School of Landscape Architecture of Beijing Forestry University, Beijing, China; United Arab Emirates University College of Medicine and Health Sciences, UNITED ARAB EMIRATES

## Abstract

Cooking methods can change the composition of foods and have important effects on human health. The Chinese people have developed many distinct and unique cooking methods. However, the daily cooking patterns of Chinese people and the characteristics and evolution of trends in cooking patterns commonly used by Chinese consumers remain unclear. The objective of this study was to identify the major cooking patterns and discuss their effects on human health, as well as to identify the cooking pattern consumer clusters and the evolution of trends in Chinese consumer cooking patterns. From March to June 2021, this study interviewed 4,710 residents in Eastern China regarding the consumption frequency of each cooking method when food is prepared at home or when eating out. Exploratory factor analysis, K-Means cluster analysis, Chi-square test, pairwise comparisons of multiple sample rates, and multivariate linear regression were used to identify the cooking patterns and cooking pattern consumer clusters, to assess differences in consumption preferences between consumer clusters, and to examine the relationship between demographic characteristic variables and different cooking patterns. Results revealed three major cooking patterns, namely traditional Chinese (cooking methods with native Chinese characteristics), bland, and high-temperature cooking patterns, as well as seven cooking pattern consumer clusters and their demographic characteristics in the Eastern Chinese population. With increases in age, education level, and income, consumers tended to choose the healthy “Bland” cooking pattern. Further, there was a higher proportion of people aged 36–65 years in the C_3_ cluster, which is characterized by the “Bland” cooking pattern. However, participants who were male and younger made fewer healthy choices in their cooking patterns. Specifically, a higher proportion of participants aged 21–35 years were found in the C_5_ cluster, which is characterized by the unhealthy “High-temperature” cooking pattern. Therefore, culinary health education should focus on individuals who are male and young. Specifically, the shift in cooking patterns among people aged 21–35 years should receive special attention.

## Introduction

Cooking is the main method of food preparation. Cooking not only modifies the organoleptic condition of foods but also influences the bioavailability of nutrients, vitamins, and minerals [1, 2]. Studies from several countries have shown that cooking patterns are associated with a higher risk of hypertension [3], inflammatory and cardio-metabolic conditions [4], and type 2 diabetes (T2D) [5]. Therefore, healthy cooking methods should be promoted.

The Chinese people have developed many distinct and unique cooking methods. Data show that there are more than 30 types of basic cooking techniques commonly used in China [6]. However, it is unclear what kinds of cooking patterns Chinese people use on a daily basis and whether these cooking patterns are good for human health. In addition, with the development of the social economy, Chinese people’s daily cooking patterns are also undergoing slight changes, but the evolution of trends in cooking patterns commonly used by Chinese consumers remain unclear. Therefore, understanding these factors and identifying the consumer clusters may lead to better guidance for consumers in adopting healthy cooking methods.

Eastern China (including Jiangsu, Shandong, Anhui, Zhejiang, and Fujian Provinces and Shanghai Municipality) lies in the east of China, and its regional cuisine occupies an important position in Chinese cuisine culture. Among the eight major cuisines in China, five of these are found in Eastern China [7]. The per capita gross regional production of East China was 103,169 Chinese yuan in 2020, higher than the national average [8]. Therefore, studies performed in East China are useful for guiding decision- and policy-making for the rest of the country. The objectives of this study were to identify the major cooking patterns and discuss their effects on human health, to identify the cooking pattern consumer clusters that exist in the Eastern Chinese population, and to clarify the evolution of trends in consumer cooking patterns.

## Methods

### Study location and sampling

**Fig 1:**
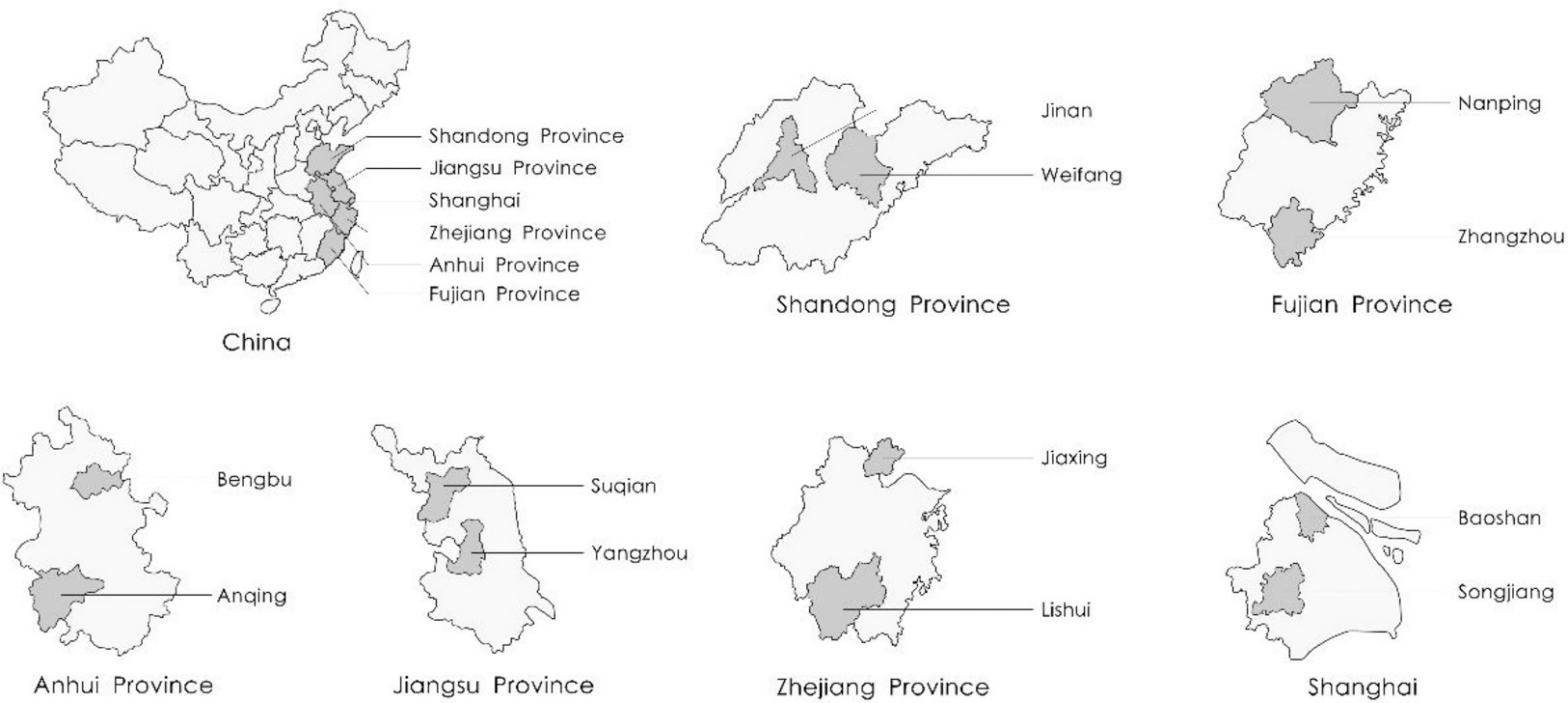
Map of the study areas in China.

The present cross-sectional study was conducted in Eastern China from March to June 2021. Potential study participants were identified using mixed stratified sampling and random sampling. Two cities were randomly selected from each province, and then two residential areas from each city were randomly selected (Fig 1). Two residential areas were randomly selected from the Shanghai Municipality, and 200 residents were recruited randomly from each residential area. Five trained research assistants conducted face-to-face interviews with the residents in each region using a standardized questionnaire. Interviews were conducted at the entrance of each residential area in the afternoon, at the end of the normal workday. The average interview lasted about 10 minutes for each participant. Upon completion of the interview, each participant received a small gift worth approximately five Chinese yuan. A total of 4,710 participants completed interviews, for a response rate of 98.1%. Only 90 residents refused to participate or did not complete the interview, citing lack of time.

### Study instrument

The questionnaire included two sections. The first section was designed to collect socio-economic information including gender, age, level of education, monthly family income, and the province or municipality of residence. The second part of the survey involved cooking methods.

Based on the Comprehensive Chinese Cooking Techniques [6] and traditional cooking techniques frequently used by residents of the surveyed provinces and cities in East China, 15 major cooking methods were selected. The methods included stir-fry and sauté (putting a small amount of oil in a pan and cooking food quickly over high heat, stirring, and turning the pan), boiling (cooking in water or liquid while the temperature is at boiling point), steaming (cooking using vapor), pan-frying (cooking food in a pan with the minimum amount of oil), roasting (cooking in an oven using hot air or radiation, which cooks the food evenly), deep-frying (cooking food in hot oil while the food is totally immersed), stewing (boiling slowly or simmering in a liquid for a long period of time at low heat), marinating in spirits (soaking food in liquor or rice wine for pickled sterilization), blanching (putting food in boiling water, turning and removing it in time, and then sautéing), poaching (cooking the food in boiling water for a short time, removing and pouring into a bowl, adding fresh clear soup, then seasoning), mixing in soy sauce (processing food into strips or sheets, putting it into boiling water or hot oil, removing it, and seasoning), stir-frying and fast-sautéing (cook small foods in boiling oil or water for about 10 seconds), simmering and keeping the shape (arranging raw materials in a neat pattern, adding the right amount of stock and condiments, gently heating until mature, and keeping the shape), deep-frying and then seasoning with sauce (frying food materials over high heat until yellow and stiff, then seasoning with sauce), and marinating in rice wine (marinating the raw materials in rice wine) [7].

Participants were asked to report their adoption and consumption frequency of each cooking method when food was prepared at home or when they ate out in the previous 12 months. The methods were evaluated on a 5-point Likert-type scale: (Never eat = 1, Eat once in a while = 2, Sometimes eat = 3, Often eat = 4, and Eat every day = 5).

### Data analysis

Three stages of data analysis were conducted. First, Cronbach’s alpha (α) coefficient was calculated to assess the internal reliability of the cooking method frequency Likert Scale questions [9]. Second, The Kaiser–Meyer–Olkin (KMO) measure of sampling adequacy and Bartlett’s test of sphericity were used to evaluate the adequacy of correlation matrices with the data [10]. Principal component factor analysis was used to derive the major cooking patterns. The factors were rotated using orthogonal transformation (varimax rotation) to achieve uncorrelated factors and greater interpretability. The number of factors retained was determined using an eigenvalue ≥ 1 [11]. The cooking method with an absolute factor loading ≥ 0.5 was considered an important contributor to this pattern [12]. Third, K-means Cluster Analysis (KCA) was conducted to determine the final cooking pattern consumer clusters using one-way ANOVA of the obtained cooking pattern factor scores and a 95% confidence level [13]. The mean values of cooking pattern factor scores of each cluster (final cluster center) were used to generalize the characteristics of each cluster (Table 3). Fourth, Chi-square tests and pairwise comparisons of multiple sample rates were used to measure the preferential differences of socio-economic information among the identified cooking pattern clusters. Finally, multivariate linear regression was employed to examine the demographic characteristic variables associated with different cooking patterns. All analyses were conducted using the IBM SPSS 26.0 software package.

## Results

### Demographic characteristics

The socio-demographic characteristics of the study participants are presented in Table 1. Of the 4,710 respondents, 56.5% were female, and most respondents were aged between 36 and 55 years (47.5%). In total, 47.3% of the study participants had an education level of undergraduate college or above. A monthly family income of between 5,000 and 9,999 Chinese yuan was reported by the highest proportion of respondents (32.9%).

**Table 1:** Respondent characteristics (N = 4710).

Characteristics	N	%
Gender		
Male	2049	43.5
Female	2661	56.5
Age		
≤20 years	885	18.8
21–35	1293	27.5
36–45	1327	28.2
46–55	908	19.3
56–65	254	5.4
66–75	34	0.7
≥76 years	8	0.2
Education		
Primary or below	92	2.0
Junior high school	655	13.9
Senior high school	905	19.2
Three-year college	830	17.6
Undergraduate college	1789	38.0
Postgraduate or above	439	9.3
Monthly family income (Chinese Yuan)		
≤5000	1301	27.6
5000–9999	1548	32.9
10000–19999	1099	23.3
20000–39999	464	9.9
40000–80000	158	3.4
≥80001	140	3.0
Residence place		
Jiangsu Province	913	19.4
Shandong Province	801	17.0
Anhui Province	953	20.2
Shanghai Municipality	618	13.1
Zhejiang Province	709	15.1
Fujian Province	716	15.2

### Cooking patterns and consumer cooking pattern clusters

Cronbach’s alpha (α) coefficient of cooking method Likert scales was 0.85, indicating a high internal consistency. Both the KMO index (0.901) and Bartlett’s test (P < 0.001) indicated that the correlation among the variables was sufficiently strong for factor analysis. Factor analysis identified three cooking patterns, and we summarized and renamed each pattern according to the characteristics of cooking techniques included in each pattern: the “Traditional Chinese” cooking pattern (cooking methods with native Chinese characteristics, including Marinated in spirits; Blanching; Poaching; Mixed in soy sauce; Stir-frying and fast-sauté; Simmer and keep the shape; Deep-fry first, then season with sauce; and Marinated in rice wine), the “Bland” cooking pattern (cooking methods in which little or no oil is used to process the food, including Stir-frying and sauté, Boiling, Steaming, and Stewing), and the “High-temperature” cooking pattern (cooking methods that make heavy use of oil or direct fire to process the food, including Pan-frying, Roasting, and Deep-frying). These patterns explained 30.043%, 15.164%, and 14.446% of the variance by cooking methods adopted, respectively. The factor loading matrices for the cooking patterns are provided in Table 2.

**Table 2:** Factor-loading matrix for the three cooking patterns.

Cooking method	Cooking patterns		
	Traditional Chinese	Bland cooking	High-temperature cooking
1. Stir-frying and sauté	-0.225	0.691	-0.015
2. Boiling	-0.034	0.838	0.047
3. Steaming	0.155	0.709	0.191
4. Stewing	0.256	0.602	0.084
5. Pan-frying	0.168	-0.151	0.732
6. Roasting	0.349	0.201	0.778
7. Deep-frying	0.285	0.065	0.815
8. Marinated in spirits	0.684	0.021	0.289
9. Blanching	0.687	0.059	0.111
10. Poaching	0.785	-0.037	0.098
11. Mixed in soy sauce	0.785	0.191	0.186
12. Stir-frying and fast-sauté	0.705	-0.115	0.229
13. Simmer and keep the shape	0.769	-0.151	0.235
14. Deep-fry first, then season with sauce	0.580	0.201	0.108
15. Marinated in rice wine	0.733	0.065	0.236
Variance explained (%)	30.043	15.164	14.446

Based on the KCA, seven cooking pattern clusters were identified. Cluster C_1_ was characterized by the “Traditional Chinese” cooking pattern (mean value = 0.047), accounting for 15.3% of the participants. Cluster C_2_ was characterized by the “Bland” (mean value = 0.924) and “High-temperature” (mean value = 1.340) cooking patterns, accounting for 10.0% of the participants. Cluster C_3_ was characterized by the “Bland” cooking pattern (mean value = 0.859), accounting for 22.2% of the participants. Cluster C_4_ was characterized by the “Traditional Chinese” (mean value = 1.440) and “High-temperature” (mean value = 0.782) cooking patterns, accounting for 16.1% of the participants. Cluster C_5_ was characterized by the “High-temperature” (mean value = 1.243) cooking pattern, accounting for 9.5% of the participants. Cluster C_6_ was characterized by the “Traditional Chinese” (mean value = 1.029) and “Bland” (mean value = 0.695) cooking patterns, accounting for 9.2% of the participants. Cluster C_7_ was characterized by no obvious cooking pattern, accounting for 17.7% of the participants (Table 3).

**Table 3:** Final cooking pattern cluster centers identified based on cooking pattern factor scores and one-way ANOVA outcomes.

Cooking patterns	Cluster						
	C_1_	C_2_	C_3_	C_4_	C_5_	C_6_	C_7_
Traditional Chinese	0.047	-0.796	-0.167	1.440	-0.379	1.029	-1.027
Bland cooking	-1.33	0.924	0.859	-0.264	-0.522	0.695	-0.294
High-temperature cooking	-0.602	1.340	-0.578	0.782	1.243	-1.153	-0.292
N (%)	719 (15.3)	472 (10.0)	1044 (22.2)	760 (16.1)	446 (9.5)	435 (9.2)	834 (17.7)

### Differences in demographic characteristics between cooking pattern clusters

There was a significantly higher proportion of female consumers in Cluster C_3_ (64.8%) compared with Cluster C_1_, Cluster C_4_, and Cluster C_5_. A significantly higher proportion of male consumers were found in Cluster C_5_ (46.4%) compared with the other clusters (χ^2^ = 82.80, P < 0.000). There was a significantly lower proportion of consumers aged ≤ 20 years in Cluster C_3_ (9.0%) compared with the other clusters, while a significantly higher proportion of consumers between the ages of 21 and 35 years was found in Cluster C_5_ (35.7%) compared with C_3_, C_6_, and C_7_. There was a significantly higher proportion of consumers between 36–45 (29.9%), 46–55 (30.3%), and 56–65 (9.3%) years in Cluster C_3_ compared with C_5_ (χ^2^ = 554.19, P < 0.000). There was a significantly higher proportion of consumers with a Junior high school education (20.7%; χ^2^ = 104.57, P < 0.000) and a monthly family income ≤ 5000 yuan (34.8%) in Cluster C_7_ compared with the other clusters. A significantly higher proportion of consumers with a monthly family income between 10,000 and 19,999 yuan was found in Cluster C_3_ (30.6%) compared with Cluster C_5_ (χ^2^ = 134.42, P < 0.000) (Table 4).

**Table 4:** Chi-square analysis of demographic characteristics found in cooking pattern clusters.

		Cluster, n (%)							Total		
		C_1_	C_2_	C_3_	C_4_	C_5_	C_6_	C_7_		χ ^2^	P-value
Gender	Male	372_a_ (51.7%)	174_b, c_ (36.9%)	368_c_ (35.2%)	393_a_ (51.7%)	207_a, d_ (46.4%)	196_d, e_ (45.1%)	339_b, e_ (40.6%)	2049 (43.5%)	82.80	0.000
	Female	347_a_ (48.3%)	298_b, c_ (63.1%)	676_c_ (64.8%)	367_a_ (48.3%)	239_a, d_ (53.6%)	239_d, e_ (54.9%)	495_b, e_ (59.4%)	2661 (56.5%)		
Age	≤20 years	135_a_ (18.8%)	107_a, b_ (22.7%)	94_c_ (9.0%)	284_d_ (37.4%)	111_b_ (24.9%)	46_c, e_ (10.6%)	108_e_ (12.9%)	885 (18.8%)	554.19	0.000
	21–35	231_a_ (32.1%)	149_a_ (31.6%)	209_b_ (20.0%)	232_a, c_ (30.5%)	159_a_ (35.7%)	93_b_ (21.4%)	220_c_ (26.4%)	1293 (27.5%)		
	36–45	203_a, b, c_ (28.2%)	123_a, b, c_ (26.1%)	312_c_ (29.9%)	159_d_ (20.9%)	106_b, d_ (23.8%)	135_a, c, e_ (31.0%)	289_e_ (34.7%)	1327 (28.2%)		
	46–55	123_a_ (17.1%)	74_a, b_ (15.7%)	316_c_ (30.3%)	69_d_ (9.1%)	54_b, d_ (12.1%)	107_e_ (24.6%)	165_a_ (19.8%)	908 (19.3%)		
	56–65	24_a_ (3.3%)	14_a_ (3.0%)	97_b_ (9.3%)	14_a_ (1.8%)	12_a_ (2.7%)	45_b_ (10.3%)	48_c_ (5.8%)	254 (5.4%)		
	66–75	3_a, b_ (0.4%)	3_a, b, c, d_ (0.6%)	14_b, d_ (1.3%)	1_a_ (0.1%)	3_a, b, c, d_ (0.7%)	8_c, d_ (1.8%)	2_a_ (0.2%)	34 (0.7%)		
	≥76 years	0_a_ (0.0%)	1_a_ (0.2%)	2_a_ (0.2%)	1_a_ (0.1%)	1_a_ (0.2%)	1_a_ (0.2%)	2_a_ (0.2%)	8 (0.2%)		
Education	Primary or below	8_a_ (1.1%)	6_a_ (1.3%)	20_a, b_ (1.9%)	12_a, b_ (1.6%)	11_a, b_ (2.5%)	10_a, b_ (2.3%)	25_b_ (3.0%)	92 (2.0%)	104.57	0.000
	Junior high school	116_a_	63_a, b_	119_b_	81_b_	46_b_	57_a, b_	173_c_	655		
		(16.1%)	(13.3%)	(11.4%)	(10.7%)	(10.3%)	(13.1%)	(20.7%)	(13.9%)		
	Senior high school	148_a, b_	68_c_	217_a, b_	170_b_	81_a, b, c_	74_a, c_	147_a, c_	905		
		(20.6%)	(14.4%)	(20.8%)	(22.4%)	(18.2%)	(17.0%)	(17.6%)	(19.2%)		
	Three-year college	135_a_	83_a, b_	186_a, b_	146_a_	88_a_	69_a, b_	123_b_	830		
		(18.8%)	(17.6%)	(17.8%)	(19.2%)	(19.7%)	(15.9%)	(14.7%)	(17.6%)		
	Undergraduate college	258_a_	187_a_	409_a_	296_a_	180_a_	168_a_	291_a_	1789		
		(35.9%)	(39.6%)	(39.2%)	(38.9%)	(40.4%)	(38.6%)	(34.9%)	(38.0%)		
	Postgraduate or above	54_a_	65_b_	93_a_	55_a_	40_a_	57_b_	75_a_	439		
		(7.5%)	(13.8%)	(8.9%)	(7.2%)	(9.0%)	(13.1%)	(9.0%)	(9.3%)		
Monthly family income (Chinese Yuan)	≤5000	202_a, b_	137_b_	214_c_	237_b, d_	120_a, b_	101_a, c_	290_d_	1301	134.42	0.000
		(28.1%)	(29.0%)	(20.5%)	(31.2%)	(26.9%)	(23.2%)	(34.8%)	(27.6%)		
	5000–9999	258_a_	157_a, b_	323_b_	230_b_	157_a, b_	137_a, b_	286_a, b_	1548		
		(35.9%)	(33.3%)	(30.9%)	(30.3%)	(35.2%)	(31.5%)	(34.3%)	(32.9%)		
	10000–19999	155_a, b, c_	100_a, b, c_	319_d_	149_c_	96_a, b, c_	113_b, d_	167_a, c_	1099		
		(21.6%)	(21.2%)	(30.6%)	(19.6%)	(21.5%)	(26.0%)	(20.0%)	(23.3%)		
	20000–39999	64_a_	40_a, b_	125_c_	81_a, c_	46_a, c_	57_c_	51_b_	464		
		(8.9%)	(8.5%)	(12.0%)	(10.7%)	(10.3%)	(13.1%)	(6.1%)	(9.9%)		
	40000–80000	22_a, b_	25_b_	33_a_	22_a_	14_a, b_	18_a, b_	24_a_	158		
		(3.1%)	(5.3%)	(3.2%)	(2.9%)	(3.1%)	(4.1%)	(2.9%)	(3.4%)		
	≥80001	18_a_	13_a_	30_a_	41_b_	13_a_	9_a_	16_a_	140		
		(2.5%)	(2.8%)	(2.9%)	(5.4%)	(2.9%)	(2.1%)	(1.9%)	(3.0%)		
Total	n	719	472	1044	760	446	435	834	4710		

^a,b,c,d^: Differences among the indicated values of a particular item were considered significant at a 95% confidence level.

### Evolution of trends in Chinese consumer cooking patterns

The results from the linear regression analysis (Table 5) showed that gender was significantly and negatively associated with the “Traditional Chinese” cooking pattern, but significantly and positively associated with the “Bland” cooking pattern. Age was significantly and negatively associated with the “Traditional Chinese” and “High-temperature” cooking patterns, and significantly and positively associated with the “Bland” cooking pattern. Education was significantly and positively associated with the “Bland” cooking pattern. Monthly family income was significantly and positively associated with both the “Traditional Chinese” and “Bland” cooking patterns.

**Table 5:** Multivariate linear regression β coefficients and standard errors for demographic characteristic variables associated with different cooking patterns (N = 4710).

	Cooking patterns					
	Traditional Chinese		Bland cooking		High-temperature cooking	
	β	SE	β	SE	β	SE
Gender	-0.089**	0.029	0.089**	0.029	-0.027	0.028
Age	-0.121**	0.012	0.183**	0.012	-0.280**	0.012
Education	0.009	0.011	0.080**	0.011	0.009	0.011
Monthly family income	0.093**	0.012	0.044**	0.012	0.014	0.012

*P < 0.05

**P < 0.01.

## Discussion

This study identified three major cooking patterns and five consumer clusters using data from 4,710 participants. Results showed that gender, age, education, and monthly family income were significantly and positively associated with the “Bland” cooking pattern, while age was significantly and negatively associated with “High-temperature” and “Traditional Chinese” cooking patterns.

The “Bland” cooking pattern was characterized by a high-frequency consumption of stir-frying and sautéing, boiling, steaming, and stewing. Steaming, boiling, and stewing mainly rely on water vapors or water to transfer heat [14]. A related study showed that boiling and sautéing, brining, and light frying tend to be cardio-metabolically beneficial [4]. The possible mechanism for this is that steaming increases the concentration of polyphenols and antioxidants [15], and dietary polyphenols have a lowering effect on LDL-C [16]. In addition, when comparing the effects of steaming, oven cooking, and deep fat frying on the physicochemical and sensory quality of turkey meat patties, steamed patties had the lowest shrinkage and fat content [17]. Thus, the “Bland” cooking pattern can be considered healthy.

The “High-temperature” cooking pattern is characterized by a high frequency of pan-frying, roasting, and deep-frying. Frying relies on oil to transfer heat, and roasting usually involves pickling on an open flame and baking by coal, firewood, or charcoal during fire roasting [14]. A previous study claimed that high-temperature cooking methods can significantly influence the nutritional quality and safety of food by analyzing breaded chicken nuggets [18]. Another study pointed out that the consumption of fried food may interact with a genetic background prone to obesity [19] and that hot barbecues produce harmful carcinogens [20]. Thus, the “High-temperature” cooking pattern can be seen as unhealthy.

The “Traditional Chinese” cooking pattern was characterized by the high-frequency consumption of different cooking methods, including marinating in spirits, blanching, poaching, mixing in soy sauce, stir-frying and fast-sautéing, simmering and maintaining the shape, deep-frying first and then seasoning with sauce, and marinating in rice wine. Although less oil is used as a medium for heating food in the abovementioned cooking methods, there have been no reports thus far indicating that these methods affect food safety or show associations with various chronic diseases. Therefore, further in-depth research is needed.

This study found that female respondents were more likely to have a higher frequency of consumption of the “Bland” cooking pattern, but males were more likely to have a higher frequency of consumption of the “Traditional Chinese” cooking pattern when they prepared food at home or when eating out. There was also a higher proportion of females in the C_3_ cluster, which is characterized by the “Bland” cooking pattern. This was consistent with a previous study that found more females chose to cook or enjoy food via stir-frying and sauté, and boiling. This may be related to their preference for beauty, emphasis on body weight, and a strong awareness of health management [21]. Interestingly, our study also found a higher proportion of males in the C_5_ cluster, which is characterized by the “High-temperature” cooking pattern, and in the C_1_ cluster, which is characterized by the “Traditional Chinese” cooking pattern. This may be because Chinese males go out to eat and socialize more than women.

This study found that respondents with higher education were also more likely to have a greater frequency of consumption of the “Bland” cooking pattern when they prepared food at home or when eating out. This was consistent with a study of Spanish adults that showed participants with greater adherence to a health-conscious pattern were more educated [4]. Our study also found that there was a higher proportion of junior high school participants in the C_7_ cluster, which had no obvious cooking pattern. Therefore, these people may be more easily guided to a healthy cooking pattern.

Our study further found that older respondents were more likely to have a higher frequency of consumption of the “Bland” cooking pattern, but younger respondents were more likely to have a higher frequency of consumption of the “Traditional Chinese” cooking pattern when they prepared food at home or when eating out. Specifically, there was a higher proportion of individuals between 36 and 65 years old in the C_3_ cluster, which is characterized by the “Bland” cooking pattern. In addition, there was a lower proportion of individuals aged ≤ 20 years in the C_3_ cluster. However, younger respondents were also more likely to have a higher frequency of consumption of the “High-temperature” cooking pattern when they prepared food at home or ate out. Specifically, there was a higher proportion of participants between 21 and 35 years old in the C_5_ cluster, which is characterized by the “High-temperature” cooking pattern. Therefore, young people should be encouraged to use healthy cooking pattern, and the focus should be on people between 21 and 35 years.

Our study also found that respondents with a higher monthly family income were more likely to have a higher frequency of consumption of the “Bland” and “Traditional Chinese” cooking patterns when they prepared food at home or when eating out. There was a higher proportion of participants with a monthly family income between 10,000 and 19,999 yuan in the C_3_ cluster. However, there was a higher proportion of participants with a monthly family income between 20,000 and 39,999 yuan in the C_5_ cluster. Therefore, this portion of the population should also be focused on.

The present study had some limitations. First, the frequency of consumption of various food groups and cooking methods was self-reported by participants and may have been influenced by memory bias. Second, the study participants were recruited in Eastern China. Therefore, the results may not be generalizable to the entire Chinese population [22]. A follow-up study is required to expand the geographical scope of the survey.

## Conclusions

This study identified three major cooking patterns, namely traditional Chinese cooking, bland cooking, and high-temperature cooking, as well as seven cooking pattern consumer clusters and their demographic characteristics in the Eastern Chinese population. Our results demonstrate that with increasing age, education level, and income, consumers tend to choose the relatively healthy “Bland” cooking pattern. However, more male and young people tend to make less healthy choices in their cooking patterns. Therefore, to reduce the impact of unhealthy cooking methods on individual health, the government should promote the healthy "Bland" cooking pattern and at the same time publicize the dangers of the “High-temperature” cooking pattern on human body, especially focusing on men and on people aged 21–35. In the meantime, nutrition researchers should make efforts to understand the impact of the “Traditional Chinese” cooking pattern on food safety and human health.

## Supporting information

S1 Checklist *PLOS ONE* clinical studies checklist. (DOTX)

S1 Appendix Questionnaire. (DOCX)
