# Supplementary material for: Promoting healthy cooking patterns in China: Analysis of consumer clusters and the evolution of cooking pattern trends
Source: PLoS One. 2023 Nov 15;18(11):e0293919. doi: 10.1371/journal.pone.0293919 (PMC10650978; doi:10.1371/journal.pone.0293919)
Supplement: S1 Appendix — (DOCX) [file pone.0293919.s002.docx]

**Appendix**

Questionnaire

**Gender**

□ Male

□ Female

**Age**

□ ≤20 years

□ 21–35

□ 36–45

□ 46–55

□ 56–65

□ 66–75

□ ≥76 years

**Education**

□ Primary or below

□ Junior high school

□ Senior high school

□ Three-year college

□ Undergraduate college

□ Postgraduate and above

**Monthly family income (Chinese Yuan)**

□ ≤5000

□ 5000–9999

□ 10,000–19,999

□ 20,000–39,999

□ 40,000–80,000

□ ≥80,001

**Province (or Municipality)**

□ Jiangsu

□ Shandong

□ Anhui

□ Shanghai Municipality

□ Zhejiang

□ Fujian

**How often do you adopt or consume each cooking method when you prepare food at home or eat out?**

| Cooking method | Never eat | Eat once in a while | Sometimes eat | Often eat | Eat every day |
| --- | --- | --- | --- | --- | --- |
| Stir-frying and sautéing | □ | □ | □ | □ | □ |
| Boiling | □ | □ | □ | □ | □ |
| Steaming | □ | □ | □ | □ | □ |
| Stewing | □ | □ | □ | □ | □ |
| Pan-frying | □ | □ | □ | □ | □ |
| Roasting | □ | □ | □ | □ | □ |
| Deep-frying | □ | □ | □ | □ | □ |
| Marinating in spirits | □ | □ | □ | □ | □ |
| Blanching | □ | □ | □ | □ | □ |
| Poaching | □ | □ | □ | □ | □ |
| Mixing in soy sauce | □ | □ | □ | □ | □ |
| Stir-frying and fast-sautéing | □ | □ | □ | □ | □ |
| Simmering and maintaining the shape | □ | □ | □ | □ | □ |
| Deep-frying first and then seasoning with sauce | □ | □ | □ | □ | □ |
| Marinating in rice wine | □ | □ | □ | □ | □ |
